# Six-Week Exercise Training With Dietary Restriction Improves Central Hemodynamics Associated With Altered Gut Microbiota in Adolescents With Obesity

**DOI:** 10.3389/fendo.2020.569085

**Published:** 2020-12-07

**Authors:** Junhao Huang, Jingwen Liao, Yang Fang, Hailin Deng, Honggang Yin, Bing Shen, Min Hu

**Affiliations:** ^1^ Guangdong Provincial Key Laboratory of Sports and Health Promotion, Scientific Research Center, Guangzhou Sport University, Guangzhou, China; ^2^ School of Basic Medical Sciences, Anhui Medical University, Hefei, China; ^3^ School of Kinesiology, Shanghai University of Sport, Shanghai, China

**Keywords:** exercise, dietary restriction, obese adolescents, gut microflora, subendocardial viability ratio

## Abstract

**Purpose:**

Obesity in children and in adolescents can lead to adult cardiovascular diseases, and the gut microbiota plays a crucial role in obesity pathophysiology. Exercise and diet interventions are typical approaches to improve physical condition and to alter the gut microbiota in individuals with obesity. However, whether central hemodynamic parameters including subendocardial viability ratio, the augmentation index standardized to a heart rate of 75/min (AIx75), resting heart rate, and blood pressure, correlate with gut microbiota changes associated with exercise and diet is unclear.

**Methods:**

Adolescents (n = 24, 12.88 ± 0.41 years) with obesity completed our 6-week program of endurance and strength exercises along with dietary restriction. Blood and fecal samples were collected, and physical parameters were measured before and 24 h after the last session of the intervention program. Pulse wave analysis using applanation tonometry provided the subendocardial viability ratio, a surrogate measure of microvascular myocardial perfusion, and AIx75, a measure of arterial stiffness and peripheral arteriolar resistance. Correlation analysis detected any associations of anthropometric or central hemodynamic parameters with gut microbiome composition.

**Results:**

Exercise and diet interventions significantly reduced body weight, body mass index, body fat, and waist-to-hip ratio, and lowered levels of fasting blood glucose, serum triglycerides, and high-density lipoprotein cholesterol. AIx75 and resting heart rate were also significantly reduced after the intervention without changes to systolic or diastolic blood pressure. The ratio of intestinal microbiota *Firmicutes* to *Bacteroidetes* displayed a marked increase after intervention. Interventional changes in gut microbiota members were significantly associated with anthropometric and metabolic parameters. Microbial changes were also significantly correlated with central hemodynamic parameters, including subendocardial viability ratio, AIx75, and resting heart rate.

**Conclusion:**

Exercise and diet interventions significantly improved measures of central hemodynamics, including subendocardial viability ratio, AIx75, and resting heart rate, which were correlated with altered gut microbiota in adolescents with obesity. Our findings shed light on the effects and mechanisms underlying exercise and diet interventions on obesity and suggest this approach for treating patients with both cardiovascular disease and obesity.

## Introduction

Obesity-related cardiovascular disease in children and adolescents is becoming more prevalent and is being accompanied by a rapid increase in childhood and adolescent obesity in wealthy societies (1). It has been shown that childhood and adolescent body mass index (BMI) is positively correlated with the risk of cardiovascular disease in adulthood, indicating that cardiovascular damage may begin during childhood (2). The intestinal microbiota has been recently suggested as an environmental factor involved in the control of body weight and energy homeostasis (3). Accumulating evidence has demonstrated that the gut microbiota plays a crucial role in obesity pathophysiology (4). The intestinal flora is dominated by two microbial phyla, *Firmicutes* and *Bacteroidetes* (5). Previous studies have shown that a higher *Firmicutes*-to-*Bacteroidetes* (F/B) ratio correlates with obesity (6).

Clinical trials have reported that a combined exercise and diet intervention improves the obesity-associated cardiovascular biomarkers of body composition, blood pressure, dyslipidemia, insulin resistance, and inflammation (7, 8). Additionally, Woo et al. have demonstrated that exercise or dietary modification may improve arterial and cardiac function (9). More recently, studies have revealed that physical exercise or diet influences the gut microbiota in individuals with obesity (10–13). In addition, Menni et al. reported that gut microbiome diversity is inversely associated with arterial stiffness in middle-aged women (14). However, it has not yet been investigated whether central hemodynamic parameters correlate with gut microbiota under the lifestyle modifications of exercise and diet.

Thus, in the present study, the effects of a 6-week program of combined endurance and strength training exercise and dietary restriction on central hemodynamic parameters and gut microbiome composition was assessed in adolescents with obesity. Furthermore, the relationship between changes in central hemodynamics and gut microbiota was determined. We hypothesized that the exercise and diet intervention would improve central hemodynamics associated with altered gut microbiota.

## Materials and Methods

### Participants and Ethical Approval

Between July and August 2018, participants with obesity who were 9 to 16 years of age were recruited from a weight loss camp after a physical examination. For inclusion, participants satisfied the obesity diagnosis criteria based on the 2004 report titled “Body mass index reference norm for screening overweight and obesity in Chinese children and adolescents” (15) with a cutoff point for obesity at 95% of the national BMI reference norm. Participants with metabolic, gastrointestinal, or cardiac diseases were excluded. None of participants were using medications known to affect energy expenditure or were losing weight in the previous 3 months whether by dieting, taking weight-loss medication, or engaging in more physical exercise than usual.

### Exercise and Diet Intervention

The full details of the exercise and diet intervention have been published in our previous studies (16, 17). In brief, participants were provided with a calorie-restricted but nutritionally complete diet based on their age. The diet consisted of balanced proportions of 60% carbohydrate, 20% protein, and 20% fat, and calories were distributed as 30%, 40%, and 30% at breakfast, lunch, and dinner, respectively. Dietitians prepared and supervised all meals. Participants performed an intense exercise program of 5 h/day, 6 days/week for 6 weeks, which was conducted from 8:00 to 9:30 am, from 10:00 to 11:30 am, and from 3:00 to 5:00 pm daily (18, 19). The exercise primarily consisted of endurance training (including bicycling, walking, running, dancing, and ball games) and strength training. The endurance exercises involved moderate- and high-intensity training. Moderate-intensity exercise was defined as 70% to 85% of the participant’s maximum heart rate (HRmax), which was calculated using the following formula: 208 − (0.7 × age). High-intensity exercise (approximately 90% of HRmax) was alternated with low-intensity exercise (approximately 60% of HRmax) during training. Strength exercise was performed at 40% to 50% of each participant’s maximal strength for two to three sets of 12 to 15 repetitions each, with 2 to 3 min rest between each set. A daily training log assessed the participants’ compliance. Measurements were performed before exercise training and 24 h after the last session of the 6-week exercise and diet program.

### Body Composition and Biochemical Factors

Height and weight were determined, and BMI was calculated as weight in kilograms divided by height in meters squared (kg/m^2^). Body composition was assessed by a body composition analyzer (Inbody 370, Biospace, Seoul, Korea). Blood samples were collected for the assessment of total cholesterol (TC), triglycerides (TG), high-density lipoprotein cholesterol (HDL-C), low-density lipoprotein cholesterol (LDL-C), and fasting glucose levels.

### Resting Heart Rate and Brachial Blood Pressure

Resting heart rate (HR), brachial systolic blood pressure (SBP), and diastolic blood pressure (DBP) were obtained using a sphygmomanometer (Omron, 705 IT, Omron Health Care, Japan). Measurements were made in triplicate, and the average value was used.

### Pulse Wave Analysis

Pulse wave analysis was noninvasively performed using applanation tonometry (SphygmoCor, AtCor Medical, Sydney, Australia) to obtain the subendocardial viability ratio (SEVR) and wave reflection data. SEVR is a surrogate measure of microvascular myocardial perfusion and was calculated as the ratio of the pressure area under the curve during diastole to the pressure area under the curve during systole. Low SEVR levels indicate reduced subendocardial perfusion. Augmentation pressure is expressed as the difference between the second and first systolic peaks of the ascending aortic waveform. The augmentation index (AIx) was assessed with the SphygmoCor device as a percentage of the ratio of augmentation pressure to aortic pulse pressure. To exclude to influence of heart rate on the AIx, AIx is normalized to a standard heart rate of 75 beats/min (AIx75). AIx75 is considered a measure of arterial stiffness and peripheral arteriolar resistance.

### Fecal Sample Collection and 16S rDNA Sequencing

To assess the intestinal flora of each participant, we collected the intestinal contents of each individual before exercise (3 days after enrollment) and after intervention (1 day after 6 weeks of the diet and exercise intervention) (20). After each defecation, the contents of the intestinal tract were placed in separate sterile fecal collection tubes (5 mL), and liquid nitrogen was immediately poured into the tubes for preservation.

Fecal samples were used to obtain total genomic DNA samples for sequencing of the 16S rRNA gene. A library for sequencing was prepared according to Illumina’s 16S Metagenomic Sequencing Library Preparation Guide. The library quality was determined using a Qubit 2.0 Fluorometer (Thermo Scientific) and an Agilent Bioanalyzer 2100 system. The Illumina MiSeq platform sequenced the hypervariable regions V3–V4 of the 16S rRNA gene and generated the raw data.

Cutadapt (version 2.8) was used to remove low-quality readings and adapter sequences from the raw data (**Figure S1**). The software FLASH (version 1.2.11) (21) is used for sequence splicing, and the paired reads obtained by pair-end sequencing are assembled into a sequence by using the overlap relationship to obtain the tags of the hypervariable region. Use the software VSEARCH (version 2.3.4) (22) to cluster the spliced Tags into operational taxonomic units (OTUs). After obtaining the OTU representative sequence, the OTU representative sequence is compared with the database for species annotation through RDP Classifier (version 2.2) software (23), and the confidence threshold is set to 0.8. The comparison database includes: Silva (version 128) (24), Greengenes (version 201305) (25), and UNITE (version 6 20140910) (26). After the spliced tags are optimized, they are clustered into OTUs for species classification with 97% similarity, and the abundance information of each sample in each OTU is counted. The Venn diagram can be used to show the number of common and unique OTUs of multiple samples, and visually show the overlap of OTUs between samples. Combined with the species represented by OTU, the core microorganisms in different environments can be found. QIIME 2 (version 2019.4) (27) calculates classification summary and alpha and beta diversity indicators, statistical analysis, and classification. R script was used for downstream analysis, including rank abundance curves (most abundant ranked 1, next most abundant ranked 2, etc.) based on operational taxonomic units (OTUs), principal component analysis (using ade4 package), a Venn diagram (using VennDiagram package), and alpha (using mothur package) and beta diversity analyses of the OTU abundance.

### Statistical Analysis

Linear discriminant analysis (LDA) effect size was used to estimate the magnitude of the impact on each taxonomic group represented by differences pre- and post-intervention. These analyses were represented by bar graphs, the parameters were set to the default *P*-value < 0.05, and the LDA score of LEfSe was 2.0 (28). Analyses were performed using SPSS 16.0 (SPSS Inc., Chicago, IL, USA). Paired-sample *t*-tests were used to compare the data obtained before and after the intervention. Pearson correlation analysis was performed to measure the association between two linear quantitative measures. Data are shown as mean ± SE. A *P*-value < 0.05 is used to indicate statistical significance.

## Results

### Effects of Exercise and Diet Intervention on Anthropometric and Metabolic Parameters

Twenty-four participants (15 males, 9 females) met the criteria for inclusion, completed the program, and provided valid measurements at both baseline and post-training.

Exercise and diet interventions may affect many parameters in our body, for example, anthropometric and metabolic parameters. As shown in **Table 1**, in response to the exercise and diet intervention, participants who had been classified with obesity had significantly reduced body weight (before: 82.64 ± 3.40 kg vs. after: 74.00 ± 3.56 kg, *P* < 0.001), BMI (before: 31.02 ± 0.94 kg/m^2^ vs. after: 27.78 ± 0.88 kg/m^2^, *P* < 0.001), body fat (before: 34.35 ± 2.05 kg vs. after: 26.30 ± 1.73 kg, *P* < 0.001), and waist-to-hip ratio (before: 0.93 ± 0.01% vs. after: 0.88 ± 0.12%, *P* < 0.001). Participants with obesity also showed significantly lower levels of fasting blood glucose (before: 4.37 ± 0.08 mmol/L vs. after: 4.14 ± 0.07 mmol/L, *P* < 0.05), serum TGs (before: 4.15 ± 0.18 mmol/L vs. after: 3.25 ± 0.07 mmol/L, *P* < 0.001), and LDL-C (before: 2.30 ± 1.14 mmol/L vs. after: 1.73 ± 0.08 mmol/L, *P* < 0.001) following the intervention. However, no significant changes in levels of serum TC (before: 0.70 ± 0.11 mmol/L vs. after: 0.76 ± 0.06 mmol/L, *P* = 0.576) or HDL-C (before: 1.15 ± 0.05 mmol/L vs. after: 1.16 ± 0.05 mmol/L, *P* = 0.790) were found (**Table 1**).

**Table 1:** Anthropometric, metabolic, and hemodynamic parameters of participants before and after 6-week combined exercise and diet intervention.

Parameter	Before (mean ± SE)	After (mean ± SE)	*P*-value
Age (year)	12.88 ± 0.41	–	–
Gender (n) (M/F)	24 (15/9)	–	–
Body weight (kg)	82.64 ± 3.40	74.00 ± 3.56	<0.001
BMI (kg/m^2^)	31.02 ± 0.94	27.78 ± 0.88	<0.001
Body fat (kg)	34.35 ± 2.05	26.30 ± 1.73	<0.001
Waist-to-hip ratio (%)	0.93 ± 0.01	0.88 ± 0.12	<0.001
Blood glucose (mmol/L)	4.37 ± 0.08	4.14 ± 0.07	0.014
Triglycerides (mmol/L)	4.15 ± 0.18	3.25 ± 0.07	<0.001
Cholesterol (mmol/L)	0.70 ± 0.11	0.76 ± 0.06	0.576
HDL-C (mmol/L)	1.15 ± 0.05	1.16 ± 0.05	0.790
LDL-C (mmol/L)	2.30 ± 1.14	1.73 ± 0.08	<0.001
SEVR (%)	112.21 ± 6.89	163.08 ± 8.61	<0.001
AIx75 (%)	9.45 ± 1.49	3.13 ± 2.18	0.006
Resting HR (beats/min)	77.08 ± 2.87	60.88 ± 2.65	<0.001
SBP (mm Hg)	114.54 ± 2.51	110.79 ± 1.85	0.079
DBP (mm Hg)	66.75 ± 1.82	63.79 ± 1.99	0.104

BMI, body mass index; HDL-C, high-density lipoprotein cholesterol; LDL-C, low-density lipoprotein cholesterol; SEVR, subendocardial viability ratio; AIx75, augmentation index standardized to a heart rate of 75/min; HR, resting heart rate; SBP, systolic blood pressure; DBP, diastolic blood pressure.

### Effects of the Exercise and Diet Intervention on Central Hemodynamics

To investigate the effects of the exercise and diet intervention on central hemodynamics, we next performed pulse wave analysis. Compared with the SEVR in normal conditions (130–200%) (29, 30), individuals with obesity had lower SEVR values (112.21 ± 6.89%). After the intervention, these individuals displayed significant augmentation in their SEVR (163.08 ± 8.61%, *P* < 0.001). A previous study reported the normal and reference value of AIx75 was 9.4 ± 7.7% for age < 34 years (31). After the intervention, these individuals displayed significant reductions in AIx75 (before: 9.45 ± 1.49% vs. after: 3.13 ± 2.18%, *P* = 0.006) and resting heart rate (before: 77.08 ± 2.87 beats/min vs. after: 60.88 ± 2.65 beats/min, *P* < 0.001) (**Table 1**). However, there were no significant changes in levels of SBP (before: 114.54 ± 2.51 mmHg vs. after: 110.79 ± 1.85 mmHg, *P* = 0.079) and DBP (before: 66.75 ± 1.82 mmHg vs. after: 63.79 ± 1.99 mmHg, *P* = 0.104) following the exercise and diet intervention (**Table 1**).

### Effects of the Exercise and Diet Intervention on Intestinal Microbiota Composition

**Figure 1:**
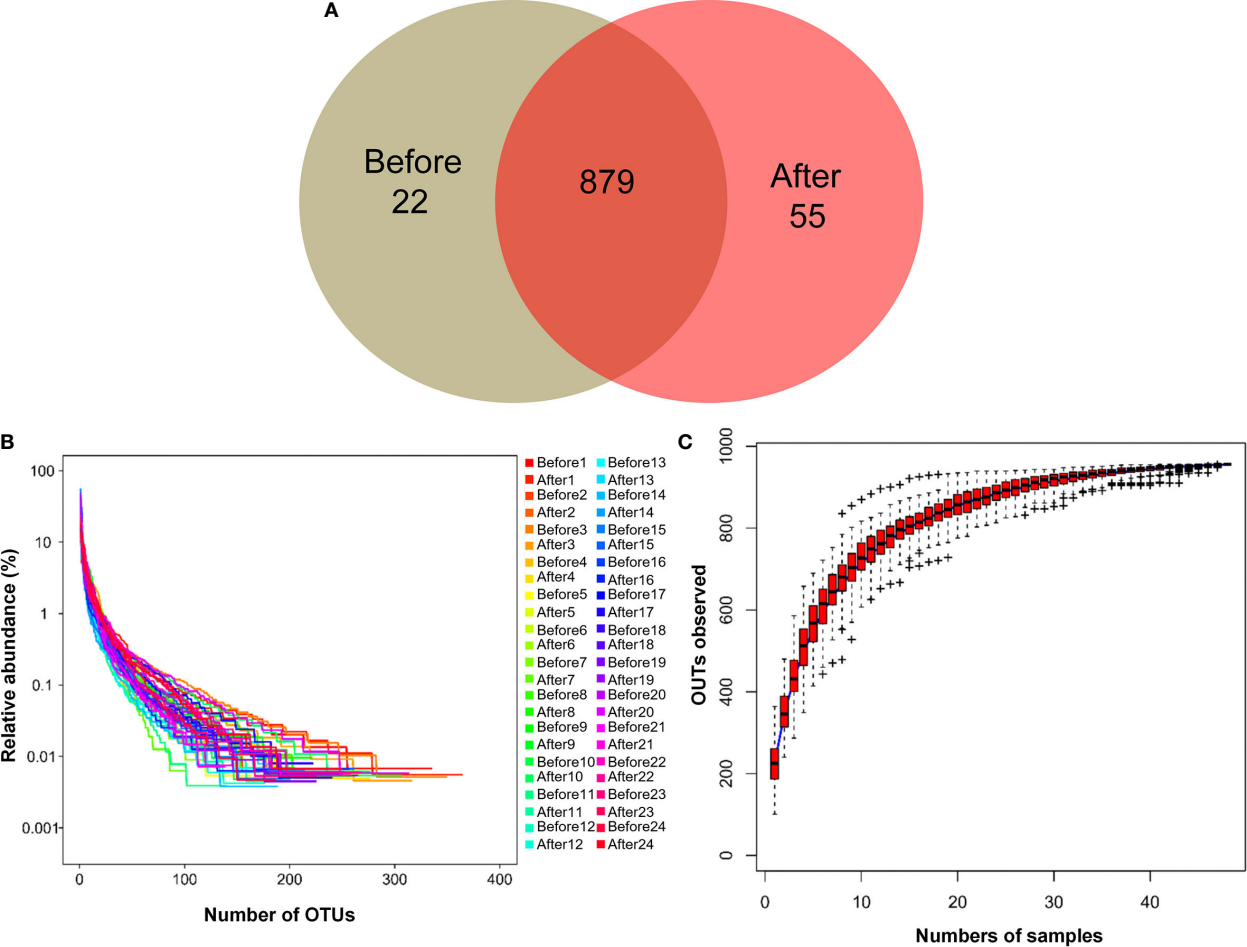
Characteristics of two microbiota. **(A)** Venn diagram. The Venn diagram shows the unique operational taxonomic units (OTUs) pre-intervention (Before; brown) and post-intervention (After; light-red), and the number of shared OTUs (dark-red) pre- and post-intervention. **(B)** OTU rank abundance curves. The legend indicates the Sample Number. Before presents pre-intervention. After presents post-intervention. **(C)** Species accumulation boxplot of the 48 samples.

To evaluate gut microbial composition, high-throughput DNA sequencing using V3-V4 16S rDNA libraries was performed with fecal DNA from participants before and after the diet and exercise intervention. We obtained a total of 1,574,058 tags, with an average of 32,792 tags per sample. There were 956 OTUs in a total of 48 samples. Pre-intervention, there were 901 OTUs, and post-intervention there were 934 OTUs. We detected 22 unique OTUs pre-intervention and 55 unique OTUs post-intervention (**Figure 1A**). The OTU rank abundance curve (**Figure 1B**) shows species diversity in a sample that simultaneously explains the richness and uniformity of the species contained in the sample. We found that each sample had good species richness and uniformity. The species accumulation boxplot of the 48 samples is shown in **Figure 1C**. As the sample size increased, no significant increase was detected in the microbial species present, indicating that the sample size used in the present study was sufficient to capture species diversity. Rarefaction, a statistical technique, was used with measures that estimate species evenness and richness across samples, the Simpson index (range, 0–1, with higher values indicating less diversity) and the Shannon index (value increasing with the number of overall species and as the distribution of species across samples becomes even). The results showed that both indices increased with sequencing and flattened as the number of sequences sampled increased (**Figure S2**), suggesting that the sequencing data were sufficient to cover nearly all microbiota species in all samples and to reveal accurate microbiological information. In addition, beta diversity was calculated, and the results were plotted in Nonmetric Multidimensional Scaling (NMDS) 3D (**Figure S3**). However, our results showed that no clustering was observed using the ANOSIM test (*P* = 0.721).

**Figure 2:**
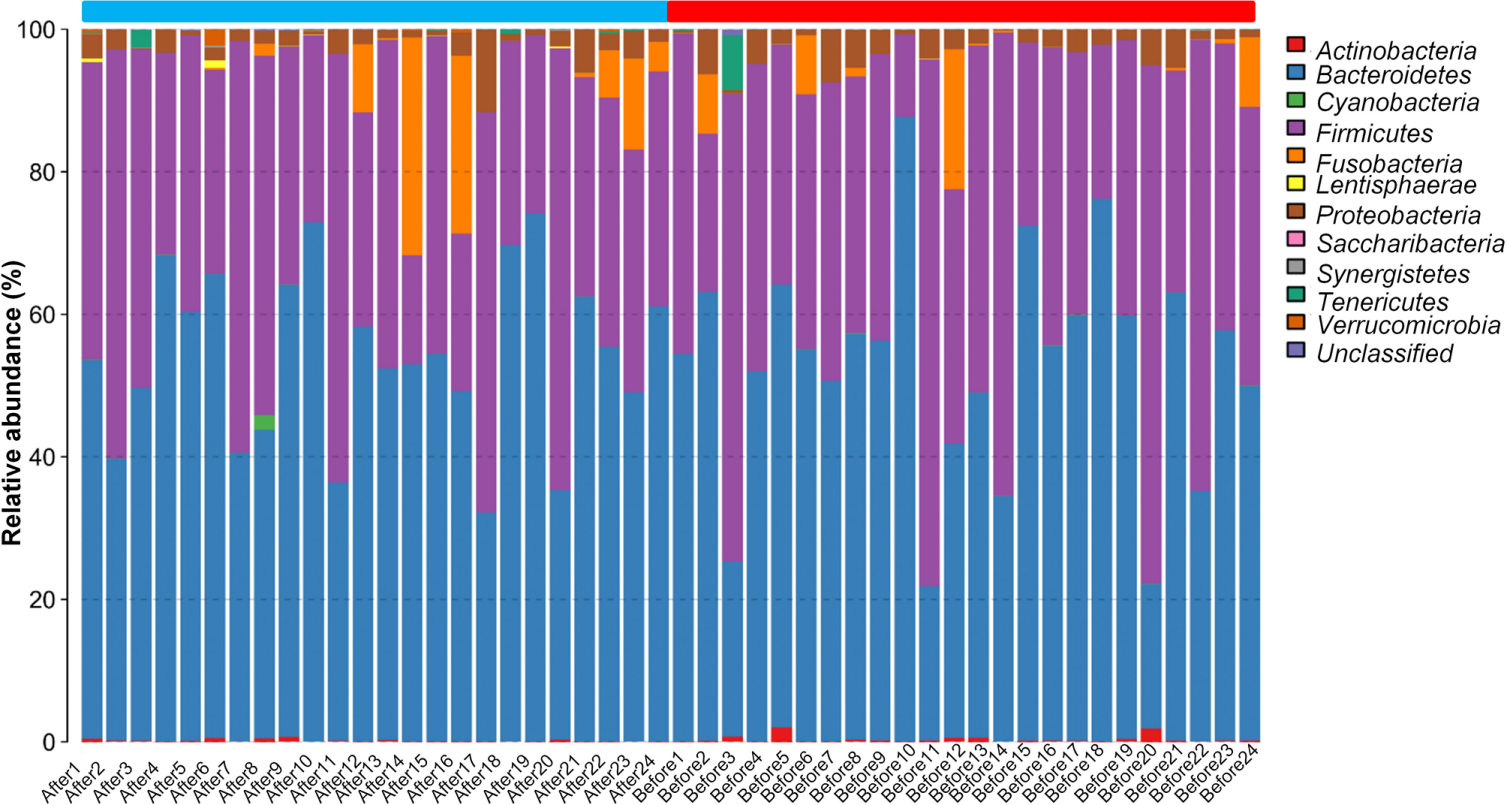
The taxonomic composition distribution in samples at the phylum level. The abscissa represents the Sample Number. Before presents pre-intervention, After presents post-intervention. The 48 samples are divided into two parts. The blue part is from After1 to After24, and the red part is from Before1 to Before24. The legend in the upper right corner represents the name of the species at the phylum level. The content of the bacteria is displayed in the histogram in the corresponding proportion and color.

We next evaluated the composition of the gut microbiota at the phylum level. The most abundant phyla were *Bacteroidetes* (53.24%) and *Firmicutes* (40.50%), followed by *Fusobacteria* (2.92%), *Proteobacteria* (2.61%), *Actinobacteria* (0.26%), *Tenericutes* (0.25%), *Verrucomicrobia* (0.08%), *Lentisphaerae* (0.04%), *Cyanobacteria* (0.04%), *Synergistetes* (0.01%), and *Saccharibacteria* (0.004%) (**Figure 2**). *Firmicutes* and *Bacteroidetes*, two major phyla of the domain *Bacteria*, are dominant in gut microbiota. Thus, the F/B ratio has been extensively examined for human gut microbiota, including to assess the flora of individuals with obesity. Numerous studies have shown that the F/B ratio is correlated with obesity and with other diseases. Therefore, we analyzed the dominant strains at the phylum level pre- and post-intervention. We found that the level of *Firmicutes* decreased whereas the level of *Bacteroidetes* increased after the diet and exercise intervention. The F/B ratio was significantly decreased after the intervention (post-intervention vs. pre-intervention: 0.737 ± 0.09 vs. 1.11 ± 0.205; n = 24 in each group; *P* = 0.011).

We further compared the alpha diversity pre- and post-intervention to assess changes in intestinal flora diversity and abundance after the intervention (**Table 2**). The average number of observed species (Sobs) was significantly increased (before: 203.59 ± 10.53 vs. after: 240 ± 12.00, *P* = 0.002), indicating that the abundance of intestinal flora post-intervention was superior to that pre-intervention. The Chao and Ace diversity indexes indicate the species richness of the samples, with higher values indicating greater species richness. The Shannon and Simpson indexes indicate the species uniformity and richness of the samples. An increased Shannon index or a decreased Simpson index represents higher population diversity. Of these estimators, we found that the Chao (before: 259.77 ± 14.15 vs. after: 299.77 ± 16.33, *P* = 0.007), Ace (before: 265.66 ± 15.33 vs. after: 298.70 ± 14.87, *P* = 0.023), and Shannon indexes (before: 2.93 ± 0.09 vs. after: 3.19 ± 0.10, *P* = 0.009) were significantly increased, whereas the Simpson index (before: 0.14 ± 0.02 vs. after: 0.11 ± 0.01, P = 0.035) was significantly decreased after the intervention.

**Table 2:** Intestinal flora diversity and abundance before and after 6-week combined exercise and diet intervention.

Alpha diversity measure	Before (mean ± SE)	After (mean ± SE)	*P*-value
Sobs	203.59 ± 10.53	240 ± 12.00	0.002
Chao	259.77 ± 14.15	299.77 ± 16.33	0.007
Ace	265.66 ± 15.33	298.70 ± 14.87	0.023
Shannon	2.93 ± 0.09	3.19 ± 0.10	0.009
Simpson	0.14 ± 0.02	0.11 ± 0.01	0.035

Sobs, observed species.

**Figure 3:**
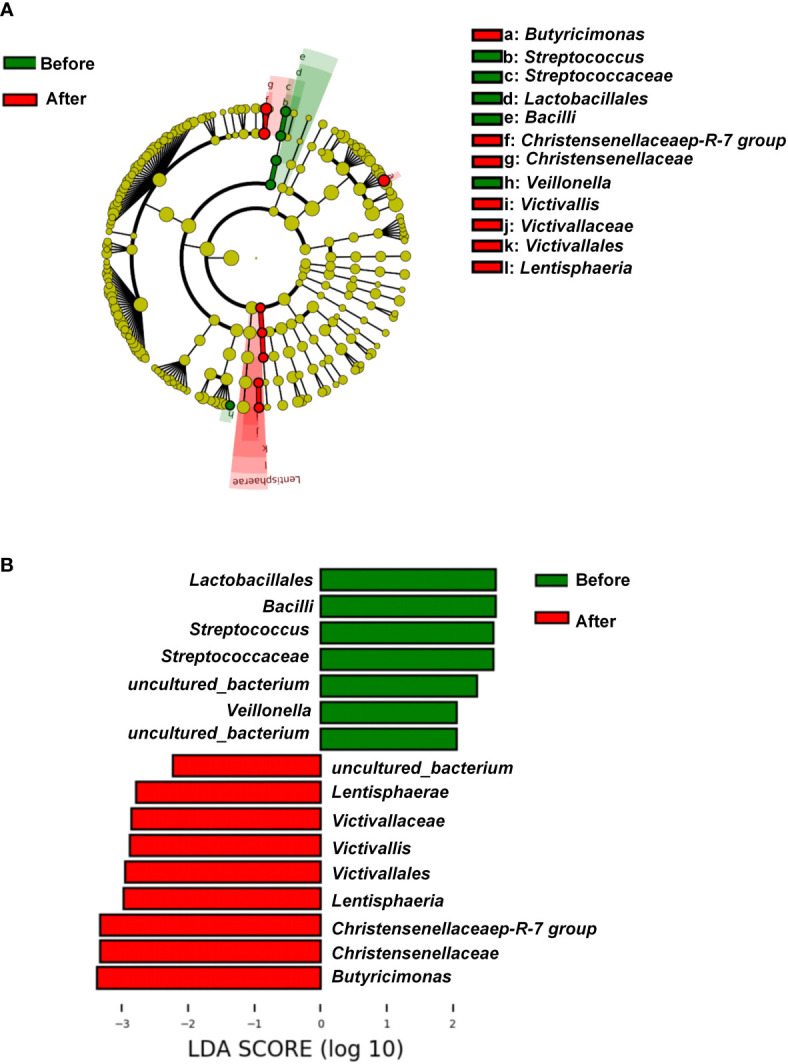
Linear discriminant analysis effect size before and after diet and exercise intervention. **(A)** Cladogram of the gastric microbial taxa associated with individuals before and after diet and exercise intervention. Taxa higher in relative abundance before the intervention are in green, and those higher after the intervention are in red. **(B)** Histogram of the linear discriminant analysis (LDA) scores for differentially abundant taxonomic features before and after the intervention. Significance obtained by LDA effect size at *P* < 0.05, (Kruskal–Wallis test) and LDA score >2.

We further explored the differentially rich taxonomic groups by using LDA effect size as a biomarker discovery algorithm. In addition to uncultured bacterium, we found 12 classified biomarkers, of which 5 were rich pre-intervention, and 7 were rich post-intervention (**Figure 3A**). The LDA scores indicated that the relative abundances of *Lactobacillales, Bacilli*, *Streptococcaceae*, *Streptococcus*, and *Veillonella* were higher pre-intervention than post-intervention (**Figures 3A, B**). These five microorganisms are all members of *Firmicutes* phylum. By contrast, the results showed higher relative abundances of *Lentisphaeria*, *Victivallales*, *Victivallaceae*, *Victivallis*, *Christensenellaceae*, *Christensenellaceae* R-7 group, and *Butyricimonas* post-intervention vs. pre-intervention (**Figures 3A, B**). These seven microorganisms all belong to the *Bacteria* domain. These results indicate that the exercise and diet intervention had a marked effect on bacteria.

### Significant Associations Between Changes in Gut Microbiota Members and Metabolic Parameters After the Exercise and Diet Intervention

**Figure 4:**
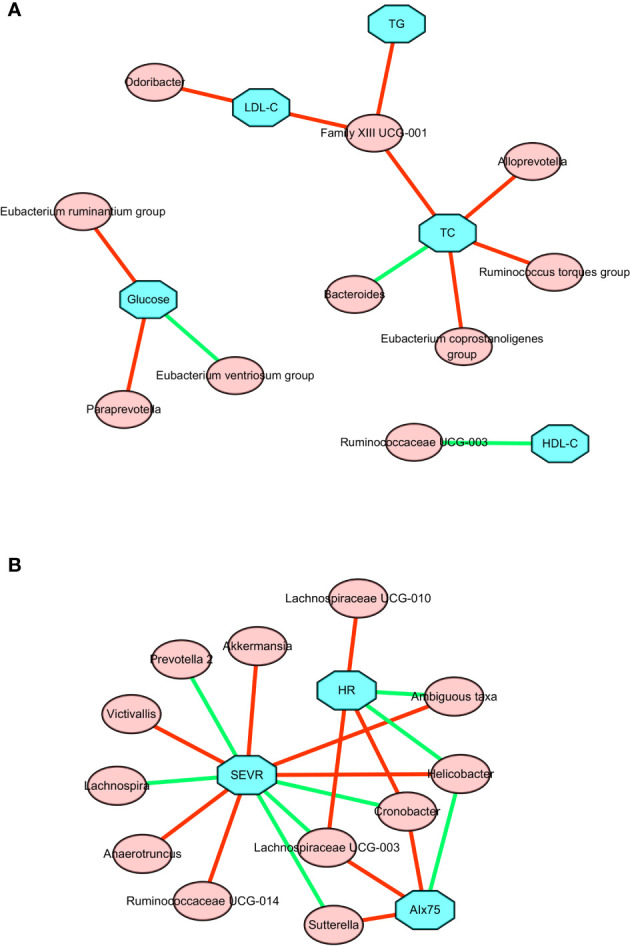
Correlation network between changes in gut microbiota members and clinical parameters after a 6-week combined exercise and diet intervention. **(A)** Correlations between changes in gut microbiota members and metabolic parameters. **(B)** Correlations between changes in gut microbiota members and central hemodynamics. The blue square represents clinical parameters, the red circle represents gut microbiota; the red line represents the positive correlation coefficient, and the green line represents the negative correlation coefficient.

As shown in **Figure 4A** and **Table S1**, changes in TGs, serum TC, and LDL-C were positively associated with change in the relative abundance of *Family XIII* UCG-001. The change in TC was also positively associated with the changes in *Alloprevotella*, *Eubacterium coprostanoligenes* group, and *Ruminococcus torques* group. By contrast, there was a negative association between the change in TC and the change in *Bacteroides*. A negative association was also found between the change in HDL-C and the change in *Ruminococcaceae* UCG-003. The change in LDL-C was positively associated with the change in *Odoribacter*. The change in fasting glucose level was positively correlated with changes in the *Eubacterium ruminantium* group and *Paraprevotella*, but negatively correlated with change in the *Eubacterium ventriosum* group. Taken together, these results suggested there is a significant correlation between improved lipid profiles and changed gut microbiota composition following the present exercise and diet intervention.

### Associations of Changes in Central Hemodynamics With Changes in Gut Microbiota Composition After the Exercise and Diet Intervention

To further explore the relationship between microbial changes and cardiovascular capacity, we performed correlation analyses of cardiovascular related indicators with all microbial flora. The associations between changes in central hemodynamics and changes in gut microbiota composition are shown in **Figure 4B** and **Table S2**. Changes in SEVR, AIx75, and resting HR were positively or negatively associated with the changes in the relative abundance of 14 gut microbiota. *Akkermansia*, ambiguous taxa, *Anaerotruncus*, *Helicobacter*, *Ruminococcaceae* UCG-014, and *Victivallis* were positively correlated with SEVR, but *Cronobacter*, *Lachnospira*, *Lachnospiraceae* UCG-003, and *Prevotella* 2 were negatively correlated with SEVR. We also observed that *Cronobacter*, *Lachnospiraceae* UCG-003, and *Sutterella* had a positive correlation with AIx75, but *Helicobacter* had a negative correlation with AIx75. *Cronobacter*, *Lachnospiraceae* UCG-003, *Lachnospiraceae* UCG-010, and the *Ruminococcaceae* NK4A214 group were positively correlated with resting HR, while ambiguous taxa, *Helicobacter*, *Roseburia*, and *Ruminococcaceae* UCG-014 were negatively correlated with resting HR. *Cronobacter*, *Lachnospiraceae* UCG-003, and *Helicobacter* were all related to SEVR, AIx75, and resting HR. These findings suggested that microbial changes may be associated with cardiovascular function changes.

## Discussion

The present study is the first, to our knowledge, to show that the central hemodynamic changes of reduced AIx75 and resting HR and increased SEVR were associated with altered gut microbiota after a 6-week exercise and dietary intervention in adolescents with obesity.

It is known that childhood and adolescent obesity are associated with an increased risk of type 2 diabetes mellitus and cardiovascular disease in adulthood. Abnormalities in arterial and cardiac function play a critical role in the increased risk of cardiovascular events in individuals with obesity (32). It has been established that obesity is closely related to vascular remodeling, arterial stiffness, and cardiac events (2, 33). Pulse wave analysis is a validated and noninvasive technique for estimating microvascular coronary perfusion (SEVR) and wave reflection as proxies of arterial stiffness and peripheral arteriolar resistance (AIx75). SEVR is a useful tool to evaluate cardiac workload, myocardial perfusion, and coronary flow reserve. A decrease in SEVR indicates impaired subendocardial perfusion (34). Studies by other groups have also shown that low SEVR correlates with impaired coronary flow reserve in patients with hypertension, type I and type II diabetes, or low cardiorespiratory fitness in central obesity (35, 36). SEVR has been investigated in individuals with obesity, and evolving evidence shows that SEVR is altered in children and adolescents with obesity, although the mechanism by which this effect is mediated has not been fully elucidated (16, 37). Recent studies showed that SEVR was positively correlated with insulin sensitivity in both obese and lean adolescents, suggesting an important role of SEVR in early cardiovascular risk determination in children and adolescents (38, 39). The results of the present study indicated that SEVR increased by approximately 53% in adolescents with obesity following a diet and exercise intervention program and that AIx75 was significantly decreased by approximately 22% after the intervention. Taken together, these findings suggest that adolescents with obesity had improved myocardial perfusion and vascular function after a 6-week program of exercise and diet intervention.

In recent years, with the in-depth study of intestinal flora, researchers have found that the intestinal flora is closely associated with obesity, coronary heart disease, hypertension, type 2 diabetes, cancer, and other diseases (40). The intestinal microorganisms that have been identified in humans are mainly composed of *Bacteroides*, *Firmicutes, Actinomycetes*, *Proteobacteria*, and *Verrucomicrobia*. *Firmicutes* and *Bacteroides* are dominant among these gut microbiota, accounting for more than 90% of the total intestinal flora (41). There is typically a complex and delicate dynamic balance relationship between the intestinal flora and the host, and the intestinal microorganisms are rich in diversity; however, obese populations have reduced diversity (42). In our experimental intervention, a total of 956 OTUs were obtained, of which 22 were unique before the intervention, and 55 were unique after the intervention. In addition, we found that the F/B ratio was decreased after the intervention compared with that before the intervention. Some studies have suggested that *Bacteroides* and *Firmicutes* are markers of obesity, with animal models showing that the F/B ratio is higher in obese than in normal weight animals (5, 43, 44). Through our analyses of bacterial diversity and species abundance, we found that 6 weeks of exercise and diet intervention not only increased the intestinal microbial diversity of adolescents with obesity but also markedly changed the abundance of their intestinal bacterial flora. Therefore, our findings suggest that exercise and diet can change the intestinal microbial diversity and bacterial flora abundance. Although evidence has suggested F/B ratio as a potential biomarker of obesity, the value of F/B ratio varies in different populations and could be affected by many lifestyle-associated factors such as diet, physical activity, food additives and contaminants, antibiotic consumption, and physical activity (45). Therefore, the specific association between F/B ratio and the obesity needs to be elucidated in future studies.

We observed that the abundance of *Lactobacillales*, *Bacilli*, *Streptococcaceae*, and *Veillonella* was significantly reduced after the exercise and diet intervention. In a study comparing the intestinal flora of patients with obesity and coronary heart disease vs patients with obesity alone, it was found that the abundance of *Lactobacillus*, *Streptococcus*, and *Veillonella* was higher in those patients with both obesity and coronary heart disease than in those with obesity alone (46). A study of the intestinal flora of Wistar Kyoto rats (WKY rats) and WKY rats with spontaneous hypertension (SHR) found that the abundance of streptococci in SHR rats was significantly higher than that in WKY rats (47). In a study examining the intestinal flora of overweight adult women, a 6-week endurance exercise program significantly reduced the abundance of streptococci (48). In our study, adolescents with obesity did not have significant hypertension or coronary heart disease, but under experimental intervention, the abundance of *Lactobacillales*, *Streptococcaceae*, and *Veillonella* was decreased significantly. This finding suggests that our experimental intervention may effectively regulate intestinal microorganisms associated with obesity-related coronary heart disease and hypertension, thereby reducing the risk of these conditions. *Christensenellaceae* is relatively low in obese phenotypes compared with non-obese phenotypes, whereas this bacteria is enriched in lean phenotypes (49–51). Our result showed that relative abundances of *Christensenellaceae* were significantly enhanced after 6 weeks of exercise and diet intervention. In the study of obese gut flora, a variety of physiological characteristics related to obesity are affected by specific gut flora (52, 53). In our study, we found that microbial changes were significantly correlated with changes in SEVR, AIx75, and resting HR. For examples, changes in *Cronobacter*, *Lachnospiraceae* UCG-003, and *Helicobacter* were all positively or negatively associated with the changes in SEVR, AIx75, and resting HR. Such results suggest that exercise and diet interventions have the potential to improve central hemodynamics in part *via* changing the intestinal flora.

A limitation of our current study is the lack of a control group during the training program of the intervention. Since the camp was located in a remote district of the city, China, and subjects enrolled in the program came from various locations across the country and had an average BMI over 30 kg/m^2^, the selection of an appropriate control group was restricted (54, 55). The lack of a designated control group allows a less clear interpretation of the results. The results observed in this study will require confirmation in randomized controlled trials. Furthermore, due to the fact that there are only 24 participants with 15 males and 9 females, we think it is difficult to present the results by sex in the current study. Future studies on sex difference in microbiota composition will be warranted.

## Conclusions

The present study showed that an exercise and diet intervention not only reduced body weight but also improved central hemodynamic measures that were associated with altered gut microbiota in adolescents with obesity. Our findings offer valuable mechanistic insights for understanding some of the effects of exercise and diet interventions on obesity.

## Data Availability Statement

The datasets presented in this study can be found in online repositories. The names of the repository/repositories and accession number(s) can be found below: NCBI BioProject, accession no: PRJNA675198.

## Ethics Statement

This study was conducted according to the Declaration of Helsinki and was reviewed and approved by the Ethics Committee of Guangzhou Sport University (approval No. GSU2017003). The trial was registered in ClinicalTrials.gov (NCT03762629). Written informed consent to participate in this study was provided by the participants’ legal guardian/next of kin. 

## Author Contributions

JH, JL, and YF carried out the experiments and analyzed the data. JH, JL, YF, HD, and HY designed a part of the study and helped to interpret part of the data. BS and MH conceived the idea, designed and supervised the study, analyzed the data, and co-wrote the paper. All authors contributed to the article and approved the submitted version.

## Funding

The current work was supported by grants from the National Natural Science Foundation of China (grants 31971105, 31771315, 31600969, 81570403) and the Project of Educational Commission of Guangdong Province of China (2017KTSCX109).

## Conflict of Interest

The authors declare that the research was conducted in the absence of any commercial or financial relationships that could be construed as a potential conflict of interest.
