# Supplementary figures and images for: Six-Week Exercise Training With Dietary Restriction Improves Central Hemodynamics Associated With Altered Gut Microbiota in Adolescents With Obesity
Source: Front Endocrinol (Lausanne). 2020 Dec 7;11:569085. doi: 10.3389/fendo.2020.569085 (PMC7750456; doi:10.3389/fendo.2020.569085)

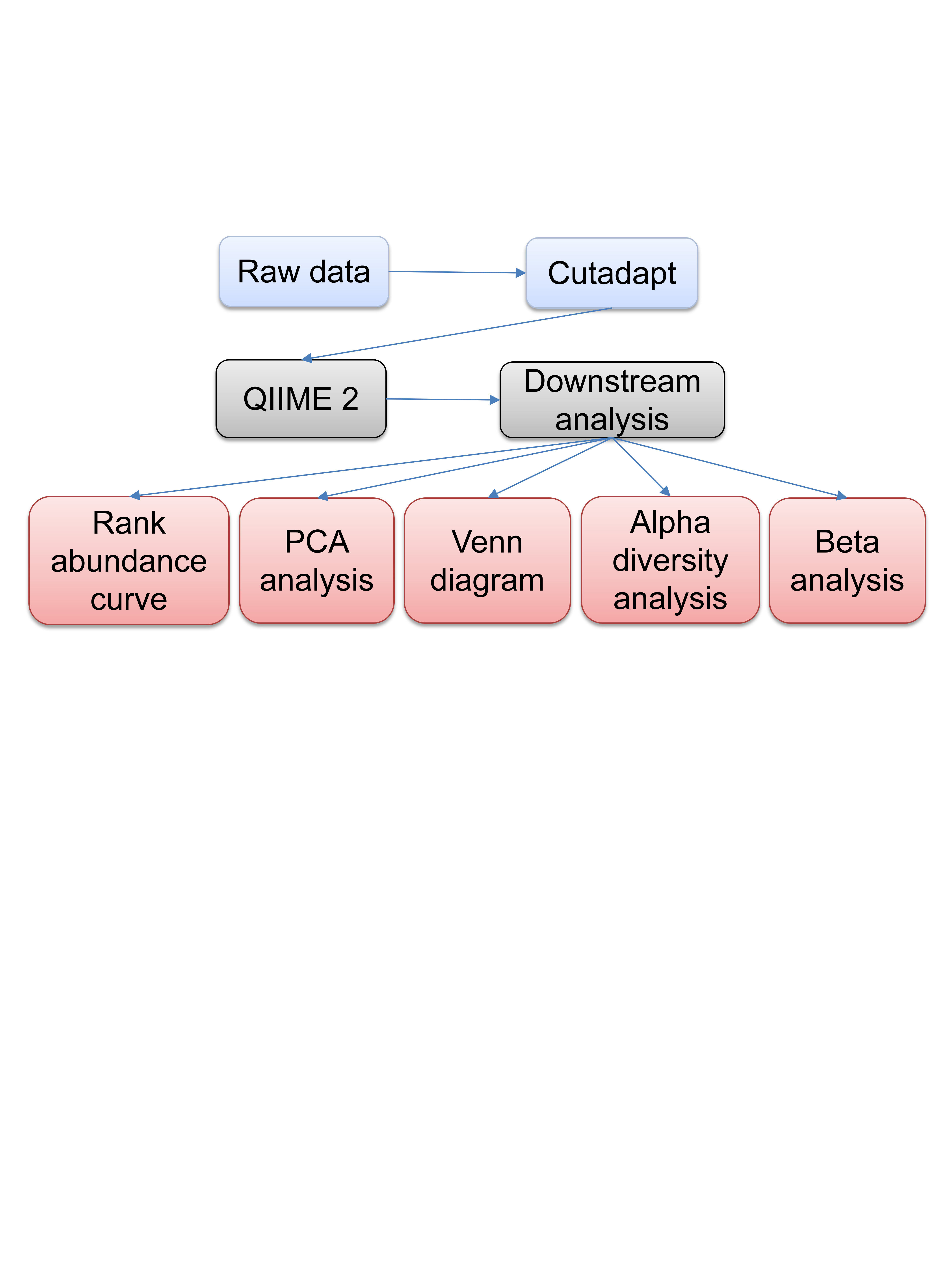

Supplement: Supplementary file 1 [file Image_1.jpeg]

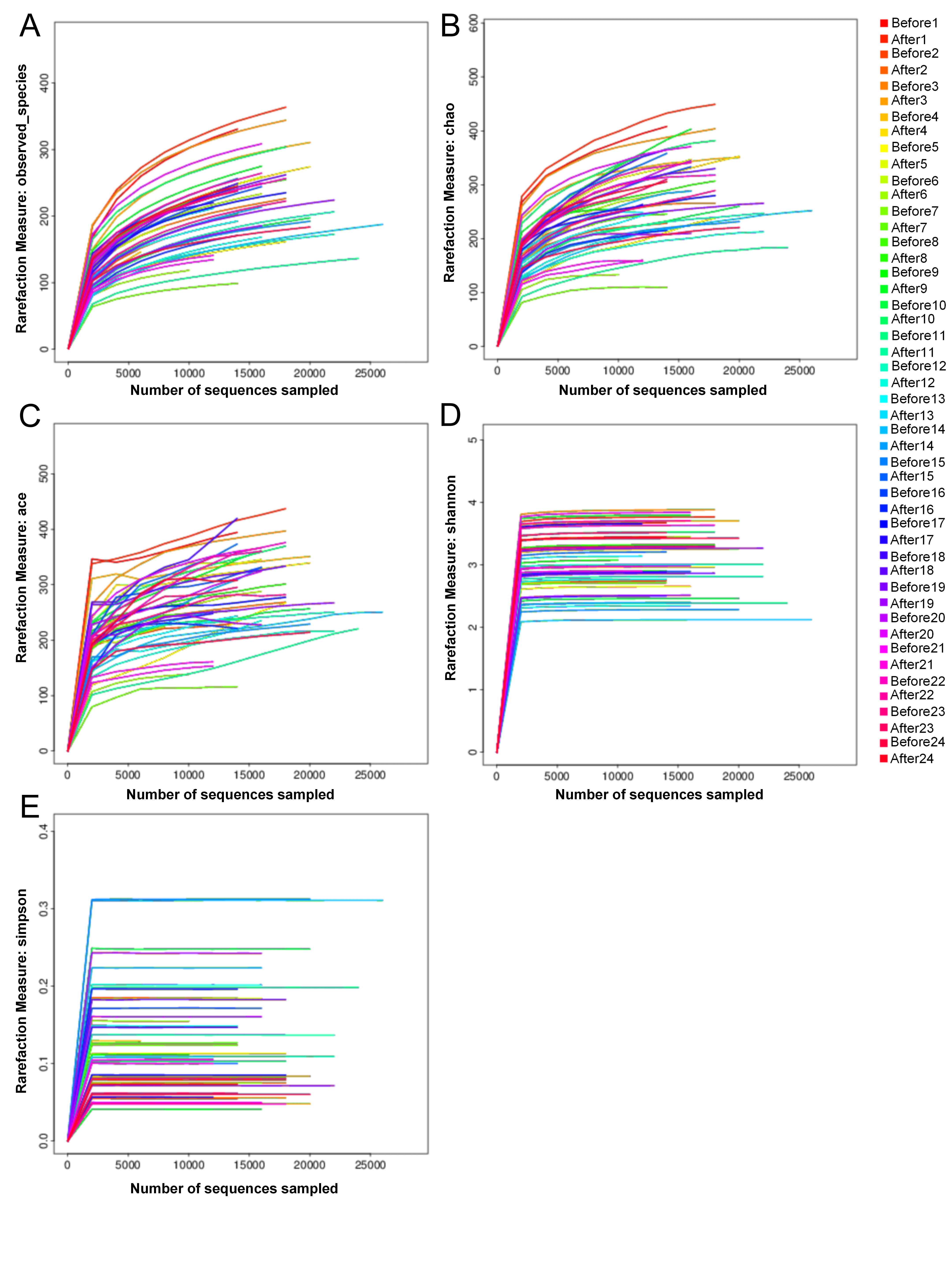

Supplement: Supplementary file 2 [file Image_2.jpeg]

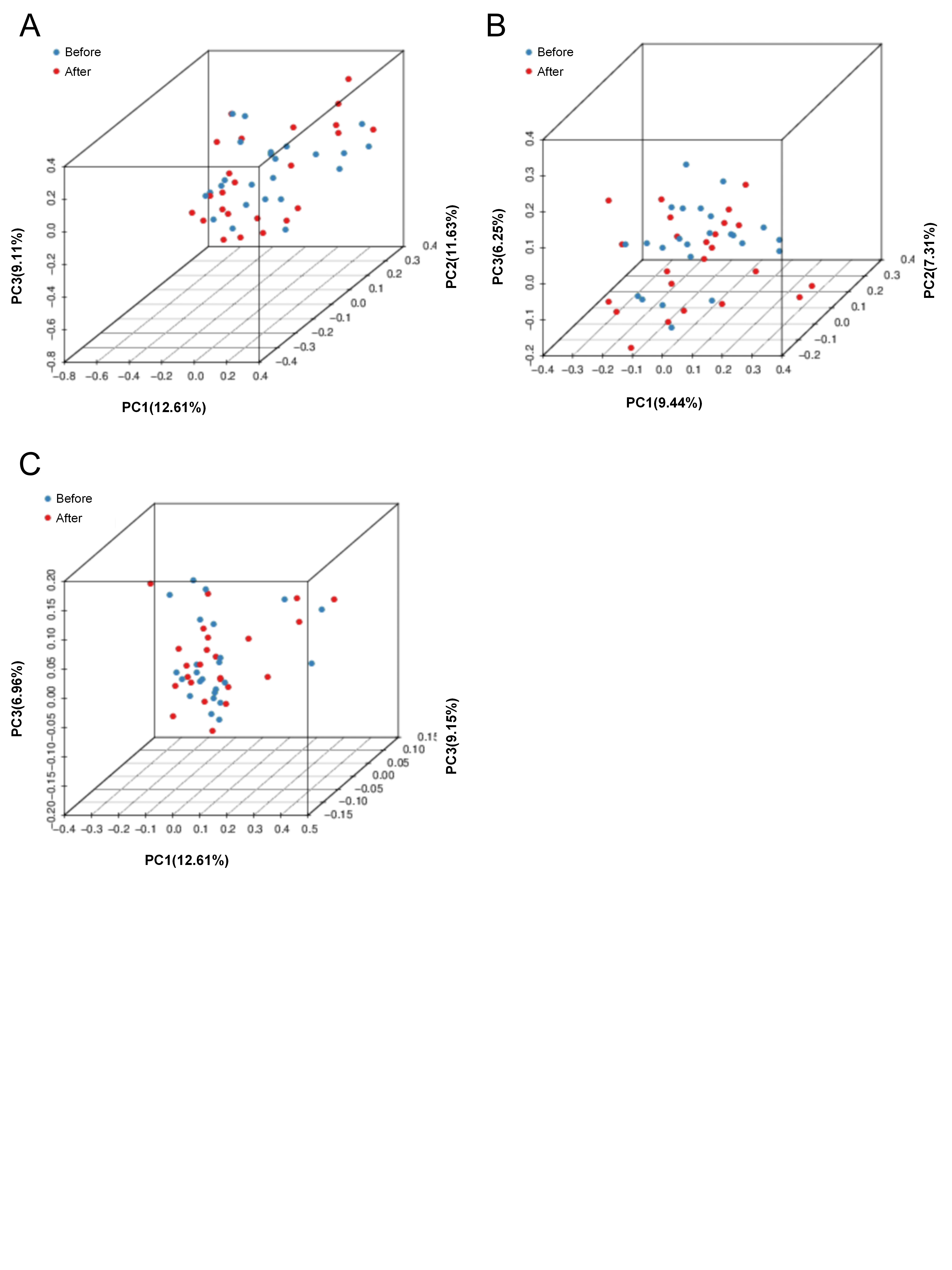

Supplement: Supplementary file 3 [file Image_3.jpeg]
