# Supplementary material for: Six-Week Exercise Training With Dietary Restriction Improves Central Hemodynamics Associated With Altered Gut Microbiota in Adolescents With Obesity
Source: Front Endocrinol (Lausanne). 2020 Dec 7;11:569085. doi: 10.3389/fendo.2020.569085 (PMC7750456; doi:10.3389/fendo.2020.569085)
Supplement: Supplementary file 4 [file Table_1.docx]

**Table S1.** **Correlations between changes in gut microbiota members and metabolic parameters after a 6-week combined exercise and diet intervention**

| Parameter | Microbiota | *P* | *r* |
| --- | --- | --- | --- |
| Glucose | *Eubacterium ruminantium* group | 0.035 | 0.432 |
| Glucose | *Eubacterium ventriosum* group | 0.004 | -0.600 |
| Glucose | *Paraprevotella* | 0.004 | 0.559 |
| TG | *Family XIII* UCG-001 | 0.013 | 0.498 |
| TC | *Alloprevotella* | 0.004 | 0.567 |
| TC | *Bacteroides* | 0.045 | -0.412 |
| TC | *Eubacterium coprostanoligenes* group | 0.026 | 0.453 |
| TC | *Family XIII* UCG-001 | 0.036 | 0.430 |
| TC | *Ruminococcus torques* group | 0.007 | 0.535 |
| HDL-C | *Ruminococcaceae* UCG-003 | 0.049 | -0.407 |
| LDL-C | *Family XIII* UCG-001 | 0.044 | 0.414 |
| LDL-C | *Odoribacter* | 0.042 | 0.417 |

Abbreviations: TG, triglycerides; TC, total cholesterol; HDL-C, high-density lipoprotein cholesterol; LDL-C, low-density lipoprotein cholesterol.
