# Supplementary material for: Six-Week Exercise Training With Dietary Restriction Improves Central Hemodynamics Associated With Altered Gut Microbiota in Adolescents With Obesity
Source: Front Endocrinol (Lausanne). 2020 Dec 7;11:569085. doi: 10.3389/fendo.2020.569085 (PMC7750456; doi:10.3389/fendo.2020.569085)
Supplement: Supplementary file 5 [file Table_2.docx]

**Table S2. Correlations between changes in gut microbiota members and central hemodynamics after a 6-week combined exercise and diet intervention**

| Parameter | Microbiota | *P* | *r* |
| --- | --- | --- | --- |
| SEVR | *Akkermansia* | < 0.001 | 0.636 |
| SEVR | Ambiguous taxa | 0.039 | 0.423 |
| SEVR | *Anaerotruncus* | 0.004 | 0.563 |
| SEVR | *Cronobacter* | 0.006 | −0.540 |
| SEVR | *Helicobacter* | 0.005 | 0.558 |
| SEVR | *Lachnospira* | 0.006 | −0.546 |
| SEVR | *Lachnospiraceae* UCG-003 | 0.005 | −0.558 |
| SEVR | *Prevotella* 2 | 0.005 | −0.555 |
| SEVR | *Ruminococcaceae* UCG-014 | 0.005 | 0.554 |
| SEVR | *Sutterella* | 0.016 | −0.486 |
| SEVR | *Victivallis* | < 0.001 | 0.650 |
| AIx75 | *Cronobacter* | 0.002 | 0.592 |
| AIx75 | *Helicobacter* | 0.002 | −0.608 |
| AIx75 | *Lachnospiraceae* UCG-003 | 0.002 | 0.610 |
| AIx75 | *Sutterella* | 0.004 | 0.567 |
| HR | Ambiguous taxa | 0.048 | −0.407 |
| HR | *Cronobacter* | 0.013 | 0.501 |
| HR | *Helicobacter* | 0.007 | −0.534 |
| HR | *Lachnospiraceae* UCG-003 | 0.007 | 0.534 |
| HR | *Lachnospiraceae* UCG-010 | 0.011 | 0.505 |
| HR | Roseburia | 0.036 | −0.430 |
| HR | *Ruminococcaceae* NK4A214 group | 0.019 | 0.476 |
| HR | *Ruminococcaceae* UCG-014 | 0.010 | −0.516 |

Abbreviations: SEVR, subendocardial viability ratio; AIx75, augmentation index standardized to a heart rate of 75/min; HR, resting heart rate.
